# The Effects of Accentuated Eccentric Loading on Mechanical Variables and Agonist Electromyography during the Bench Press

**DOI:** 10.3390/sports8060079

**Published:** 2020-05-29

**Authors:** Alexis H. Castro, Dylan Zangakis, Gavin L. Moir

**Affiliations:** Department of Exercise Science, East Stroudsburg University of Pennsylvania, East Stroudsburg, PA 18301-2999, USA; acastro5@live.esu.edu (A.H.C.); dzangakis2@po-box.esu.edu (D.Z.)

**Keywords:** resistance training, kinematics, kinetics

## Abstract

We compared the effects of accentuated eccentric loading (AEL) on mechanical variables and agonist muscle activation using low (30% 1-repetition maximum (1RM)) and high (80% 1RM) upward-phase loading with AEL (100% 1RM during downward phase) to traditional loading schemes (T) in the bench press. Twelve resistance-trained men (26 ± 6 years; 1RM: 134 ± 33 kg) performed sets of two repetitions with three-minute intervals using loading schemes of 30AEL, 30T, 80AEL, and 80T. AEL was applied using weight releasers while force plates and a 3D motion-analysis system were used to measure mechanical variables. Electromyographic activity of the pectoralis major and triceps brachii muscles was also recorded. The greater downward-phase loads experienced during the AEL conditions allowed greater overall mean vertical forces (mean difference ($\bar{x}$_Diff_): 118 N, *p* < 0.001), greater work ($\bar{x}$_Diff_: 43 J, *p* < 0.001), and greater pectoralis major muscle activation ($\bar{x}$_Diff_: 27 µV, *p* = 0.002) compared to the corresponding traditional loading schemes. However, there was little evidence of potentiation of the mechanical variables or muscle activity during the subsequent upward phases caused by the AEL schemes. It is possible that the use of weight releasers may disrupt lifting technique particularly during low AEL schemes thereby diminishing any benefits.

## 1. Introduction

The acute mechanical, metabolic, and hormonal responses to resistance training exercises have been proposed to drive neuromuscular adaptations that support the long-term enhancement of muscular strength and power [1,2,3]. Furthermore, the specific responses to a resistance training regime are influenced by the selection of programming variables including the load, volume, rest periods, frequency of sessions, exercise selection, and order, as well as the muscle action used [4]. Typical resistance training exercises used to develop muscular strength and power, such as the back squat and bench press, incorporate the combination of eccentric actions followed by concentric actions associated with the stretch shortening cycle as the load is lowered and then raised during each repetition. It is known that the acute mechanical, neural, metabolic, and hormonal characteristics differ between eccentric muscle actions and those observed during concentric actions; specifically, eccentric muscles actions are associated with greater tension development, greater cortical excitability despite lower motor unit activity, reduced energy per unit work, greater upregulation of satellite cells, and possibly larger IGF-I responses compared to concentric muscle actions [5]. However, the selection of loads used during typical resistance training exercises is based upon the concentric capabilities of the athlete (i.e., the load that can be raised during a given exercise). This constraint on load selection has led to the proposal that practitioners should endeavor to utilize methods to sufficiently overload the downward phase of resistance training exercises where the muscles are assumed to be operating eccentrically so that the adaptations in muscular strength and power are maximized [6,7,8].

There are a variety of methods available to overload the downward phase of resistance training exercises including tempo eccentric training, flywheel inertial training methods, plyometric training, and accentuated eccentric loading methods [9,10]. Of these, accentuated eccentric loading (AEL) methods whereby the load applied during the downward phase exceeds that experienced during the subsequent upward phase of the exercise would appear to be the most practical to implement with athletes, particularly through the use of weight-releasers. Doan et al. [11] previously observed that supramaximal AEL (105% 1-repetition maximum (1RM) during the downward phase) administered through weight releasers resulted in a greater maximal load lifted during the subsequent upward phase of the bench press exercise. These authors proposed that application of AEL had enhanced the SSC via mechanisms including increased neural stimulation, greater recovery of strain potential energy, alterations in the length of the contractile machinery, and a greater preload. However, subsequent researchers reported decrements in the force generated during the upward phase when applying accentuated eccentric loads (equivalent to 105%, 110%, and 120% of 1RM) with loads equivalent to 100% 1RM during the upward phase of the bench press exercise [12]. Despite the potential for fatigue associated with the administration of the supramaximal AEL protocol used by Ojasto and Häkkinen [12] to confound the findings, other researchers have failed to demonstrate enhancements when employing an AEL protocol with a load equivalent to 95% 1RM during the downward phase and a load equivalent to 85% 1RM during the upward phase of the bench press exercise [13]. Furthermore, Ojasto and Häkkinen [12] were able to demonstrate enhanced power output during the upward phase when using individualized submaximal AEL protocols incorporating a load equivalent to approximately 77% 1RM applied during the downward phase with loads equivalent to 50% 1RM during the upward phase, which was accompanied by greater agonist muscle activation during the upward phase of the bench press. The authors theorized that the increases in power were as the result of alterations of the stiffness of the musculotendinous elastic components due to the accentuated loads applied during the downward phase. Therefore, it would appear that consideration needs to be given to both the loads selected during the downward as well as the upward phases of the exercises when utilizing AEL protocols. However, the selection of loads used during the upward phase of the bench press exercise by previous researchers (50%, 85% and 100% 1RM) do not reflect the range of loads recommended to enhance power output (30–100% 1RM) [14].

Few of the previous investigators have detailed the mechanical differences during the downward phase of the bench press exercise, limiting their analyses to the upward phase and only measuring vertical variables (typically vertical velocity). Previous authors have stipulated that AEL protocols should impose minimal interruption to the natural mechanics of the exercise to provide an effective stimulus [10] and so an analysis of the mechanical variables during the downward and upward phases of the bench press exercise during AEL protocols would appear warranted. It is possible that the action of the weight releasers during the lift could adversely affect the technique during the movement, particularly during an exercise performed with free weights and during protocols where the difference between the loads applied during the downward and upward phases is large, thereby diminishing the enhancement of the musculotendinous units resulting from the accentuated eccentric load. Indeed, Ojasto and Häkkinen [12] noted alterations in the bench press technique between the submaximal AEL and traditional loading protocols although their analysis was limited to the action of the elbow joint. Therefore, the purpose of the present study was to investigate the differences in mechanical output and muscle activation between AEL (using a load equivalent to 100% 1RM during the downward phase) and traditional loading schemes during the bench press exercise performed with low (30% 1RM) and high (80% 1RM) loads during the upward phase. Based upon the findings of previous studies, we hypothesized that only the low AEL protocol would result in enhanced mechanical output and muscle activation during the upward phase of the exercise.

## 2. Materials and Methods

### 2.1. Study Design

The present study employed a cross-sectional design to compare the mechanical and agonist muscle activation differences between two AEL (30AEL and 80AEL) and two traditional loading schemes (30T and 80T) during the bench press exercise performed with free weights on a flat bench. The loads for 30AEL and 80AEL were 30% and 80% 1RM, respectively, for the upward phase with a constant load of 100% 1RM applied during the downward phase using mechanical weight releasers. The traditional loading sets used 30% (30T) and 80% (80T) 1RM throughout the downward and upward phases. The two loads used during the upward phases of the exercise reflect the range of loads recommended to improve muscular power output [14] as well as the loads used in previous investigations of the mechanical characteristics of the bench press exercise [15]. Two repetitions were performed under each loading condition with full recovery provided between and the mechanical and muscle activation data were averaged across the two repetitions.

### 2.2. Participants

Twelve resistance-trained men (age: 25.8 ± 6.1 years; height: 1.74 ± 0.09 m; body mass: 86.0 ± 12.5 kg; 1RM bench press: 134 ± 33 kg) volunteered to participate in this study, which was approved by the Institutional Research Board of East Stroudsburg University. Inclusion criteria for participation in the study was a 1RM in the bench press of 1.2 times body mass, a minimum of one-year resistance training at least 3 times per week, and being free from musculoskeletal injury for the 6 months prior to data collection. Each participant that met the inclusion criteria signed an informed consent prior to any testing session. None of the participants had any previous experience using weight releaser devices during the bench press exercise.

### 2.3. Procedures

Each participant attended five testing sessions across a three-week period. The 1RM for each participant was assessed in the first testing session using the two-point method, outlined by Garcia-Ramos et al. [16]. The participants were required to maintain contact between the bench and the upper back and the bench and the lower back throughout each repetition, while the feet were elevated and placed on plates attached to the bench (this was a requirement for the force platforms used in the subsequent testing sessions). The barbell was lowered to the level of the sternum and was required to touch the chest at the completion of the eccentric phase (although ‘bouncing’ the barbell from the chest was not permitted) at which point it was to be lifted as fast as possible to the full extension of the elbows. Spotters were positioned on either side of the barbell and followed its trajectory throughout each repetition without touching the barbell. After completing the lifts associated with the two-load method of 1RM determination, each participant was familiarized with the use of the mechanical weight releasers (Lifting Large, Warrenton, OR, USA), completing approximately eight repetitions with downward–upward load combinations equivalent to 30–15%, 60–30%, 80–30%, and 80–60% 1RM. The height of the releasers was adjusted such that the devices released from the barbell when it was approximately 0.15 m above the participants’ chest during the downward phase of the lift and repetitions were performed using loads that would approximate those used in the subsequent testing sessions. The participants were encouraged to execute each repetition as naturally as possible throughout these familiarization trials, not to pause at the bottom of the movement, and to perform the upward phase of the lift as fast as possible. The participants were told to grasp the bar using their typical grip width and this was measured and was used during all subsequent testing sessions.

Following at least 48 hours after the 1RM testing session, the participants were required to return to the laboratory and perform their first of the four different loading schemes (30AEL, 80AEL, 30T, and 80T), the order of which was counterbalanced across the participants. Each of these testing sessions was separated by a minimum of 48 hours and the participants were required to avoid any vigorous upper-body resistance training exercises during this time and to maintain their normal diet. A standardized warm-up comprising 10 min of dynamics stretches was completed at the beginning of each testing sessions prior to performing four sets of the bench press (10 repetitions with the unloaded barbell, six repetitions with 30% 1RM, four repetitions with 50% 1RM, and two repetitions with 80% 1RM) separated by 2 min. The barbell was then loaded with the requisite masses associated with the 30AEL, 80AEL, 30T, or 80T protocols and two lifts were completed by the participants with 3 min of passive recovery between each repetition.

During both of the repetitions performed under each of the four loading conditions the participants were required to bring the barbell to the chest in a controlled manner (i.e., not dropping the barbell and bouncing it from the chest) before lifting it as fast as possible to a position with the elbows fully extended. Spotters were placed at either end of the barbell throughout each repetition and were able to assist with the removal and return of the barbell to the rack during the 30T and 80T protocols. The spotters assisted the participants with the removal of the barbell from the rack during the 30AEL and 80AEL protocols and they ensured that the weight releaser devices were stationary before the lift commenced during the time that the participant was required to maintain the barbell in a locked-out position. However, it was ensured that the spotters did not interfere with the release of the devices from the barbell. The weight releasers contacted a raised area comprising wooden boards (totaling 4 cm in thickness) covered with layers of foam (totaling 10 cm in thickness) during the downward phase to initiate the release from the barbell. These raised areas were implemented in order to minimize the vibrations associated with the release of the devices that may interfere with the signal recorded from the force platforms. The participant was required to remain on the lifting bench between the two repetitions.

### 2.4. Collection and Calculation of Mechanical Data

The lifting bench was placed on two force platforms (Kistler Type 9286AA. Kistler, Amherst, NY, USA; 1000 Hz) to allow the recording of the ground reaction forces (GRF) during each repetition. An 8-camera 3-dimmensional motion analysis system (Vantage cameras, Vicon, Oxford, UK; 200 Hz) that was synchronized with the force platforms was used to record kinematic data associated with a retroreflective marker placed in the middle of the barbell. The 3-dimmensional reference frame that was used defined three axes as vertical (perpendicular to the laboratory floor), anteroposterior (along the length of the lifting bench), and mediolateral (orthogonal to the anteroposterior axis). The raw position data associated with the retroreflective marker were smoothed using a generalized cross-validated quintic spline procedure prior to analysis using the Nexus software (version 2.5, Vicon, Oxford, UK). The initiation of the downward phase of each repetition was established as the first negative vertical velocity of the barbell marker with the phase ending when the vertical velocity first became positive. The upward phase of each repetition was defined as the event between the first appearance of positive vertical velocity of the barbell marker and the time of the maximum vertical position attained by the barbell marker. The following mechanical variables were then calculated:

#### 2.4.1. Vertical Displacement

The position of the retroreflective marker placed on the barbell along the vertical axis during each repetition was recorded from the motion analysis system and the vertical displacement of the barbell during the downward and upward phases was calculated. The values during the downward phase were transformed to positive for ease of interpretation.

#### 2.4.2. Duration of Downward and Upward Phases

The duration of the downward and upward phases of each repetition was determined from the motion analysis data using the vertical displacement of the retroreflective marker placed in the center of the barbell.

#### 2.4.3. Mean Vertical Velocity

The position of the retroreflective marker along the three axes was differentiated with respect to time using the first central difference method to provide the velocity of the barbell. The velocity along the vertical axis was averaged during the downward and upward phases of each repetition to provide mean vertical velocity (the values during the downward phase were transformed to positive values for ease of interpretation).

#### 2.4.4. Mean Vertical Acceleration

The position of the retroreflective marker along the vertical axis was double differentiated with respect to time using the second central difference method to provide the acceleration of the barbell. The positive vertical acceleration towards the end of the downward phase of the exercise was averaged to provide the mean vertical acceleration during the downward phase. This value represents the average rate of change in vertical velocity as the barbell descended to the lowest point of the lift, reflecting the application of vertical force to the load. The positive vertical acceleration from the beginning of the upward phase until the first negative acceleration of the barbell was also averaged to provide the mean vertical acceleration during the upward phase. The value reflected the application of force to the load to initiate the upward motion of the barbell.

#### 2.4.5. Mean Vertical Force

The forces collected from the two force platforms were summed along the vertical axis to provide the reaction to the vertical force applied to the barbell during each repetition. The weight of the lifting bench and that of the participant were removed from the vertical component of the GRF by collecting a separate trial with the participant lying stationary on the bench at the beginning of each testing session. The vertical force was then averaged during the downward and upward phases to provide the mean vertical force.

#### 2.4.6. Downward Rate of Force Development

The average rate of the rise in the vertical component of the GRF was calculated during the period of positive vertical acceleration of the barbell towards the end of the downward phase of the exercise as the ratio of the change in vertical force to the change in time. The rate of force development (RFD) value represents the average rate at which the vertical force was applied to the barbell as it descended to the lowest point of the lift. Note that the corresponding RFD was not calculated during the period of positive vertical acceleration observed during upward phase of the repetitions as the vertical force applied to the barbell decreased during this period.

#### 2.4.7. Mean Mechanical Power Output

The instantaneous barbell velocities along the vertical, anteroposterior, and mediolateral axes were multiplied by the instantaneous forces along the corresponding axes during each repetition to provide the instantaneous mechanical power output. These were then integrated using the trapezoid rule to provide the work performed along each of the three axes. The work was then summed along all axes to provide total mechanical work, which was then divided by the duration of each phase to provide the mean mechanical power output during the downward and upward phases of each repetition (the values during the downward phase were transformed to positive for ease of interpretation).

#### 2.4.8. Mechanical Work

The instantaneous mechanical power output along the vertical, anteroposterior, and mediolateral axes was integrated using the trapezoid rule to provide the mechanical work performed along the axes during each repetition. The work performed along each axis was summed and the mechanical work performed during the downward and upward phases of each repetition. 

### 2.5. Collection and Calculation of Electromyographic Data

The electromyographic signal from the pectoralis major muscle and the long head of the triceps brachii muscle on the dominant arm (as determined by the participant) was recorded using a wireless system (Ultium®, Noraxon, AR, USA; 2000 Hz). Data from the anterior deltoid muscle was also collected but due to sampling problems it was not possible to analyze this data for a number of the participants. The signals were synchronized with the motion analysis system. The skin was prepared and bipolar electrodes (11 mm diameter with a 20 mm interelectrode distance) were placed according to previous recommendations [17]. The data was analyzed in myoMUSCLE® software (version 3.12, Noraxon, AR, USA) during the downward and upward phases of each repetition (determined from the motion analysis data). The digital signals were band-pass filtered (10–250 Hz) before the root mean square (RMS) were calculated over 100 ms windows. The RMS values were then averaged between the times associated with the downward and upward phases to provide the average activation for each muscle.

### 2.6. Statistical Analysis

All statistical analyses were performed using the IBM SPSS software package (version 25.0. International Business Machines Corp., Armonk, NY, USA). Measures of central tendency and spread of data were represented as means and standard deviations. Statistical differences in the average downward RFD between the loading schemes were assessed by a one-way ANOVA with repeated measures. The differences in the remaining mechanical variables and average agonist muscle activation were assessed using a three-way ANOVA with repeated-measures (Scheme, 2-levels: AEL; traditional × Load, 2-levels: low conditions with 30% 1RM; high conditions with 80% 1RM × Lifting phase, 2-levels: downward; upward). Alpha was set at *p* ≤ 0.05. Pairwise comparisons with Bonferroni corrections were used to establish where any significant differences occurred, while the 95% confidence limits (95% CL) of the differences were calculated as were the effect sizes (ES) using Cohen’s *d*. The ES were interpreted as: ≤0.19 = trivial; 0.20–0.49 = small; 0.50–0.79 = medium; 0.80–1.29 = large; ≥1.30 = very large [18].

## 3. Results

The results for the kinematic variables are shown in Table 1. The significant main effects and interactions associated with the statistical analyses are also shown in this table.

### 3.1. Vertical Displacement

There was a significant Scheme × Lifting phase interaction (*p* < 0.001) that was caused by the increase in vertical displacement from the downward to the upward phase during the AEL schemes being greater than the increase during the traditional schemes ($\bar{x}$_Diff_: 0.03 m; 95% CL: 0.02–0.05 m; ES: 1.91). A significant load × lifting phase interaction (*p* < 0.001) was due to the vertical displacement being greater under the low load conditions (30AEL and 30T) compared to the high load conditions ($\bar{x}$_Diff_: 0.03 m; 95% CL: 0.02–0.04 m; ES: 1.89). A significant scheme × load × lifting phase interaction was also revealed (*p* = 0.009). This was caused by the vertical displacement during the downward phase of 30T being greater than 30AEL ($\bar{x}$_Diff_: 0.02 m; 95% CL: 0.01–0.03 m; ES: 0.63). For the upward phase, the vertical displacement during 30T was greater than 80T ($\bar{x}$_Diff_: 0.04 m; 95% CL: 0.02–0.05 m; ES: 0.75) while that during 30AEL was greater than 80T and 80AEL ($\bar{x}$_Diff_: 0.03–0.05 m; 95% CL: 0.02–0.07 m; ES: 0.80–1.08).

**Figure 1:**
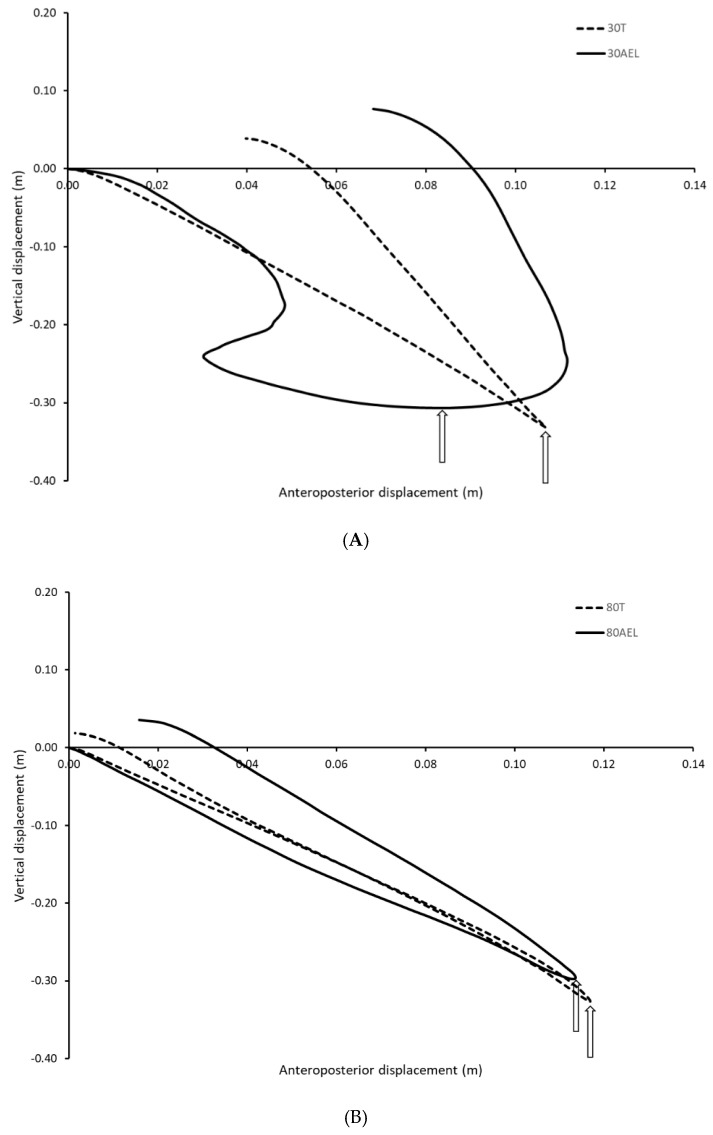
(**A**) Group averages of the two-dimensional barbell trajectory in the sagittal plane during the 30T (load during the downward and upwards phases equivalent to 30% 1-repetition maximum) and 30AEL loading scheme (load during the downward phase equivalent to 100% 1-repetition maximum; load during the upward phase equivalent to 30% 1-repetition maximum) of the bench press. Note: The downward phase of the repetition starts at the intersection of the axes; the arrows denote the approximate transition between the downward and upward phases of the lift. (**B**) Group averages of the two-dimensional barbell trajectory in the sagittal plane during the 80T (load during the downward and upwards phases equivalent to 80% 1-repetition maximum) and 80AEL loading scheme (load during the downward phase equivalent to 100% 1-repetition maximum; load during the upward phase equivalent to 80% 1-repetition maximum) of the bench press. Note: The downward phase of the repetition starts at the intersection of the axes; the arrows denote the approximate transition between the downward and upward phases of the lift.

The 2-dimmensional position of the barbell in the sagittal plane under the four loading conditions are shown in Figure 1. It can be seen that the trajectory of the barbell during the 30AEL condition was very different from that observed under the 30T loading scheme with greater displacement occurring along the anteroposterior axis, particularly during the lowest position in the repetitions (Figure 1A). There were smaller differences noted when comparing the sagittal plane trajectories for the heavy load conditions, although there was slightly greater displacement along the anteroposterior axis during the 80AEL scheme compared to 80T (Figure 1B).

### 3.2. Duration of Downward and Upward Phases

There was a significant scheme × load interaction for the duration of the downward and upward phases (*p* = 0.005). This was due to the 0.48 s increase in the durations of the phases in 30AEL compared to 30T being less than the 0.08 s increase in the durations from 80T compared to 80AEL ($\bar{x}$_Diff_: 0.40 s; 95% CL: 0.15–0.64 s), although the ES was medium (0.72). A significant load × lifting phase interaction (*p* = 0.001) was caused by the 0.51 s decrease in the duration of the upward compared to the downward phase observed during the low load conditions (30AEL and 30T) being greater than the 0.19 s reduction observed during the high load conditions ($\bar{x}$_Diff_: 0.32 s; 95% CL: 0.16–0.49 s; ES: 0.84).

A significant scheme × load × lifting phase interaction was found for the duration of the downward and upward phase (*p* < 0.001). Further analyses revealed that the duration of the upward phase during 30T was less than that during both 80AEL and 80T ($\bar{x}$_Diff_: 0.50–0.58 s; 95% CL: 0.43–0.66 s; ES: 5.77–6.24). Finally, the duration of upward phase during 30AEL was less than that during 80AEL and 80T ($\bar{x}$_Diff_: 0.46–0.54 s; 95% CL: 0.39–0.60 s; ES: 5.21–5.71).

**Figure 2:**
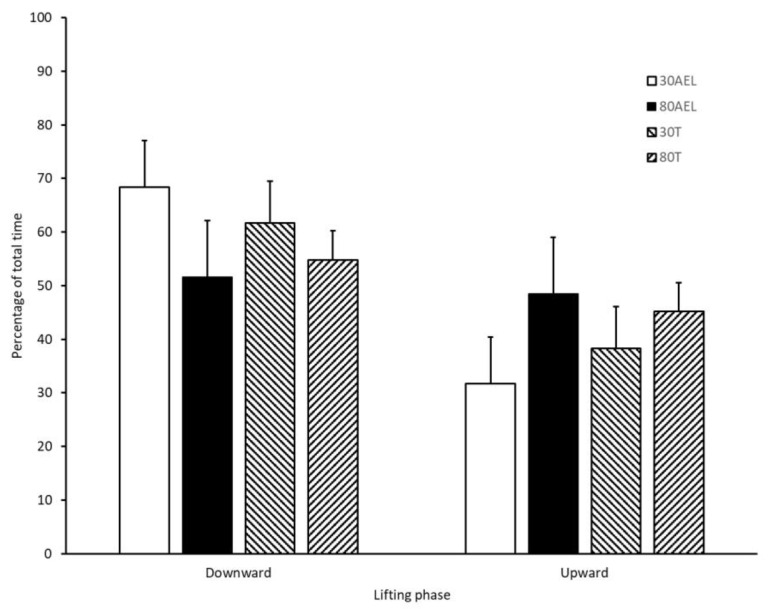
The percentage of each repetition comprising of the downward and upward phases of the bench press performed under the four different loading schemes. Note: 30AEL is the accentuated eccentric loading protocol using a load equivalent to 100% 1-repetition maximum (1RM) during the downward phase and a load equivalent to 30% 1RM during the upward phase; 80AEL is the accentuated eccentric loading protocol using a load equivalent to 100% 1RM during the downward phase and a load equivalent to 80% 1RM during the upward phase; 30T is the traditional loading protocol using a load equivalent to 30% 1RM during the downward and upward phases; 80T is the traditional loading protocol using a load equivalent to 80% 1RM during the downward and upward phases.

The percentage of each repetition comprising of the downward and upward phases under the four different loading schemes are presented in Figure 2. Both low load conditions (30AEL and 30T) produced downward phases that comprised of a greater percentage of the overall repetition time while the lifts performed under the high load conditions (80AEL and 80T) produced downward and upward phases that approximated 50% of the total repetition time.

### 3.3. Mean Vertical Velocity

A significant scheme × load interaction (*p* = 0.004) for mean vertical velocity was caused by the 0.11 m/s greater velocities attained during 30T compared to 30AEL schemes being greater than the difference observed between the 80AEL and 80T schemes ($\bar{x}$_Diff_: 0.12 m/s; 95% CL: 0.05–0.19 m/s; ES: 1.31). There was also a significant load × lifting phase interaction (*p* < 0.001) caused by the 0.47 m/s increase from the downward to the upward phases observed under the low load conditions (30AEL and 30T) being greater than the 0.06 m/s increase observed under the high load conditions ($\bar{x}$_Diff_: 0.41 m/s; 95% CL: 0.33–0.49 m/s; ES: 3.14). 

There was a significant scheme × load × lifting phase interaction for mean vertical velocity (*p* = 0.002). Specifically, the mean vertical velocity during the downward phase of 30T was greater than that during 30AEL, 80T, and 80AEL ($\bar{x}$_Diff_: 0.18–0.23 m/s; 95% CL: 0.08–0.34 m/s; ES: 0.87–1.41). For the upward phase, the mean vertical velocity during 30T was greater than that during 80T and 80AEL ($\bar{x}$_Diff_: 0.55–0.56 m/s; 95% CL: 0.44–0.66 m/s; ES: 4.11–4.35), while the mean vertical velocity during the upward phase of 30AEL was greater than that during 80T and 80AEL ($\bar{x}$_Diff_: 0.51–0.52 m/s; 95% CL: 0.41–0.62 m/s; ES: 3.67–3.89). 

### 3.4. Mean Vertical Acceleration

A significant scheme × lifting phase interaction was found for the mean vertical acceleration (*p* = 0.007). This was caused by the 0.82 m/s^2^ increase in the positive vertical acceleration from the downward to the upward phases during the 30T and 80T schemes being different from the 0.72 m/s^2^ decrease from the downward to the upward phases observed under the AEL conditions ($\bar{x}$_Diff_: 1.54 m/s^2^; 95% CL: 0.09–2.99 m/s^2^; ES: 0.96).

The results for the kinetic variables are shown in Table 2. The main effects associated with the statistical analyses are also shown in this table.

### 3.5. Mean Vertical Force

There was a significant scheme × load interaction for mean vertical force (*p* < 0.001) caused by the 181 N higher force produced during the 30AEL compared to the 30T schemes being greater than the 54 N difference observed between the 80AEL and 80T schemes ($\bar{x}$_Diff_: 127 N; 95% CL: 79–174 M; ES: 1.83). A significant scheme × lifting phase interaction (*p* < 0.001) was produced by a greater reduction in force between the downward and upward phases under the AEL schemes compared to the traditional schemes ($\bar{x}$_Diff_: 233 N; 95% CL: 158–308 M; ES: 2.88), while a significant load × lifting phase interaction (*p* < 0.001) was caused by a greater reduction in force from the downward to the upwards phases of the low load conditions (30AEL and 30T) compared to the high load conditions ($\bar{x}$_Diff_: 124 N; 95% CL: 81–168 N; ES: 1.91).

There was a significant scheme × load × lifting phase interaction for mean vertical force (*p* < 0.001). This was caused by the mean force during the downward phase of 30T being less than that during 30AEL, 80AEL, and 80T ($\bar{x}$_Diff_: 360–748 N; 95% CL: 239–886 N; ES: 1.85–3.45) while the mean vertical force during the downward phase of 30AEL was lower than both 80AEL and 80T ($\bar{x}$_Diff_: 280–388 N; 95% CL: 221–450 N; ES: 1.09–1.40). The mean vertical force during the downward phase of 80AEL was greater than 80T ($\bar{x}$_Diff_: 109 N; 95% CL: 77–141 N) although the ES was small (0.40). During the upward phase, the mean vertical force was lower in the 30T scheme compared to both 80AEL and 80T ($\bar{x}$_Diff_: 639–640 N; 95% CL: 531–749 N; ES: 3.37) while the mean force during 30AEL was lower than that during 80AEL and 80T ($\bar{x}$_Diff_: 637 N; 95% CL: 527–747 N; ES: 3.36–3.37).

### 3.6. Downward Rate of Force Development

There were no statistically significant main effects or interactions identified for the average downward RFD despite greater values being produced under the AEL schemes (*p* > 0.05; ES: 0.03–0.22). 

### 3.7. Mean Mechanical Power Output

A significant load × lifting phase interaction (*p* = 0.020) was caused by the 156 W increase in mean power output from the downward phase to the upward phase under the low load conditions (30AEL and 30T) being greater that the 74 W increase observed under the high load conditions ($\bar{x}$_Diff_: 82 W; 95% CL: 16–148 N; ES: 0.79).

### 3.8. Mechanical Work

A significant scheme × load interaction (*p* = 0.001) was caused by the 120 J greater amount of work performed during 30AEL compared with 30T being substantially more than the difference of 52 J observed between the 80AEL and 80T schemes ($\bar{x}$_Diff_: 68 J; 95% CL: 33–103 J; ES: 1.36) while a significant scheme × lifting phase interaction (*p* < 0.001) resulted from the reduction in work between the downward and upward phases of the AEL schemes (30AEL and 80AEL) being different from the increase across the phases observed under the two traditional schemes ($\bar{x}$_Diff_: 64 J; 95% CL: 38–89 J; ES: 2.59). A significant load × lifting phase interaction (*p* < 0.001) was caused by the 29 J reduction in work from the downward to the upward phases during the low load conditions (30AEL and 30T) being different from the 17 J increase in work observed under the high load conditions ($\bar{x}$_Diff_: 46 J; 95% CL: 25–66 J; ES: 2.14).

A significant scheme × load × lifting phase interaction was found for the mechanical work performed during the repetitions (*p* < 0.001). Specifically, less work was performed during the downward phase of 30T compared to 30AEL, 80AEL, and 80T ($\bar{x}$_Diff_: 113–251 J; 95% CL: 72–301 J; ES: 1.80–3.30). Less work was also performed during the downward phase of 30AEL compared to both 80AEL and 80T ($\bar{x}$_Diff_: 101–137 J; 95% CL: 81–162 J; ES: 1.17–1.46), while that performed during the downward phase of 80T was less than that during 80AEL ($\bar{x}$_Diff_: 37 J; 95% CL: 24–49 J) with a small ES (0.38). For the upward phase, more work was performed under 30AEL compared to 30T ($\bar{x}$_Diff_: 7 J; 95% CL: 3–10 J) although the ES was small (0.20), while more work was performed during the upward phase of 80T compared to 30AEL and 30T ($\bar{x}$_Diff_: 211–217 J; 95% CL: 164–264 J; ES: 2.76–2.84). Finally, more work was performed during the upward phase of 80AEL compared to 30AEL and 30T ($\bar{x}$_Diff_: 226–233 J; 95% CL: 181–278 J; ES: 3.01–3.08).

### 3.9. Muscle Activation

The data for the average activation of the pectoralis major and triceps brachii muscles are shown in Table 3. Statistically significant main effects and interactions are noted in the table. 

For the pectoralis major, a significant main effect for scheme (*p* = 0.002) was caused by the AEL (30AEL and 80AEL) schemes producing greater average activation compared to the traditional schemes. A significant main effect for Load (*p* = 0.001) was caused by the high load conditions (80AEL and 80T) producing greater average activation compared to the low load conditions (30AEL and 30T), and a significant main effect for Lifting phase (*p* = 0.002) was caused by greater average activation during the upward phases compared to the downward phases.

A significant scheme × load interaction ( *p*= 0.005) for the average activation of the pectoralis major muscle was caused by the 42 µV higher activation during 30AEL compared to the 30T scheme being greater than the 13 µV difference observed between the 80T to the 80AEL schemes ($\bar{x}$_Diff_: 29 µV; 95% CL: 11–48 µV; ES: 1.04). There was also a significant scheme × lifting phase interaction (*p* = 0.020) for the average activation of the pectoralis major muscle. This was caused by the increase in activation from the downward to the upward phase recorded under the two AEL schemes (30AEL and 80AEL) being lower than the difference recorded under the two traditional schemes ($\bar{x}$_Diff_: 51 µV; 95% CL: 10–92 µV), although the ES was small (0.43).

There was a significant main effect of load (*p* = 0.004) for the triceps brachii muscle that was caused by the high load conditions (80AEL and 80T) producing greater average activation compared to the low load conditions (30AEL and 30T); a significant main effect for the lifting phase (*p* = 0.001) was caused by greater average activation during the upward phases compared to the downward phases.

A significant scheme × lifting phase interaction was found for the average activation of the triceps brachii muscle (*p* = 0.007). This was due to the increase in the activation from the downward to the upward phases during the traditional schemes (30T and 80T) being greater than that recorded during the AEL schemes ($\bar{x}$_Diff_: 107 µV; 95% CL: 36–178 µV), although the ES was medium (0.68). Furthermore, a significant scheme × load × lifting phase interaction (*p* = 0.050) was caused by the increase in the average activation from the downward to the upward phase during 30AEL being less than the increase recorded during 80T ($\bar{x}$_Diff_: 197 µV; 95% CL: 81–312 µV; ES: 1.13).

## 4. Discussion

The purpose of the present study was to investigate the differences in mechanical output and muscle activation between AEL and traditional loading schemes during the bench press exercise performed with low (30% 1RM) and high (80% 1RM) loads during the upward phase. We hypothesized that only the low AEL protocol would result in enhanced mechanical output and agonist muscle activation during the upward phase of the exercise. Our hypothesis was not supported despite the greater magnitudes of the differences in mechanical variables reported in comparisons between 30AEL and 30T, with the comparisons of 80AEL with 80T revealing only small effect sizes. The differences identified were largely confined to the downward phase of the repetitions. For example, the mean vertical force applied to the barbell was greater during the 30AEL scheme compared to that during 30T only during the downward phase of the lift ($\bar{x}$_Diff_: 360 N; ES: 1.85). While the same effect was found in the comparison of 80AEL with 80T ($\bar{x}$_Diff_: 109 N) the ES was small (0.40). However, the mean vertical forces applied to the barbell during the upward phases of the lifts were not significantly different between the AEL and traditional schemes performed with the same upward loads. Previous researchers have reported a decrement in the force during the upward phase when applying supramaximal accentuated loads during the downward phase (equivalent to 105%, 110%, and 120% 1RM) with loads equivalent to 100% 1RM during the upward phase of the bench press exercise [12]. These same researchers also noted that upward force was not potentiated when employing submaximal AEL schemes (50–50% 1RM, 60–50% 1RM, 70–80% 1RM, 80–50% 1RM and 90–50% 1RM for downward–upward loads). However, such a finding is to be expected as the mean vertical force applied to the barbell during the upward phase of the bench press must be equal to the weight of the barbell given the phase ends with no vertical velocity (i.e., the repetition is not performed ballistically). The acceleration of the barbell towards the end of the downward phase and that during the initial period of the upward phase were included in the present study to provide further details concerning the application of force. It was found that both AEL schemes actually inhibited the generation of large vertical acceleration during the upward phase of the lift compared to the traditional loading schemes.

The greater amounts of mechanical work performed during both of the AEL schemes compared to the traditional schemes observed in the present study was a result of the greater work performed during the downward phases due to the greater loading, with the significant increase of 7 J in the work performed on the barbell during the upward phase between 30AEL and 30T producing only a small ES (0.20). This small increase was unlikely to be due to changes in the work done to raise the barbell given similar vertical forces and vertical displacements during the upward phase under the 30AEL and 30T conditions. This leaves an increase in the work performed on the barbell along the horizontal axis as the likely explanation of the small difference observed between the 30AEL and 30T schemes. Although previous researchers have not assessed the effects of AEL on mechanical work, Sheppard and Young [19] reported greater displacement of the barbell during the flight phase of the bench throw exercise using three submaximal AEL protocols (60–40 kg, 70–40 kg, and 80–40 kg for the downward–upward loads) compared to traditional loading scheme (40–40 kg downward–upward loads). Given the work-energy theorem, the greater displacement reported by these researchers is likely due to more work being performed on the barbell during the upward phase of each repetition leading to a greater kinetic energy of the load at the end of the phase (barbell release), resulting in a greater displacement during flight. An explanation for the contrasting findings may be the lower loads during the downward phase used by Sheppard and Young [19] compared to those used in the present study (e.g., loads equivalent to 60–80% 1RM vs. 100% 1RM used in the present study) or the nature of the exercise (e.g., ballistic vs. non-ballistic). We could therefore conclude that neither AEL protocol used in the present study produced a substantial enhancement of mechanical output during the upward phase of the repetitions.

Our hypothesis that the low AEL protocol would result in enhanced agonist muscle activation during the upward phase of the exercise was not supported. An analysis of the pectoralis major and triceps brachii muscles revealed that the load conditions exerted a greater effect on the average activation of these agonists compared to the AEL schemes, with greater activation associated with the high load conditions (80AEL and 80T). Previous authors have also reported limited effects of supramaximal and submaximal AEL protocols on muscle activation [12,13]. Indeed, van den Tillar and Kwan [13] actually reported evidence of a submaximal AEL protocol involving a load equivalent to 95% 1RM during the downward phase with a load equivalent to 85% 1RM during the upward phase producing muscular fatigue in a number of upper-body muscles, particularly early during the upward phase. It was noted in the present study that both of the AEL schemes actually resulted in a greater reduction in average activation of the pectoralis major and triceps brachii muscle between the downward and upward phases of the lift compared to the traditional schemes. These differences likely reflect the contrasting loads applied during the downward and upward phases of the AEL schemes as opposed to fatigue of the agonist muscles. However, it is worth noting that the activity of the antagonistic muscles was not recorded in the present study. It is possible that the activity of the antagonistic muscles, such as biceps brachii and posterior deltoid, may have altered as a result of the AEL schemes, thereby requiring lower activation of the agonists during the upward phases. Alternatively, the activity of the antagonistic muscles may have been potentiated by the heavy loads applied during the downward phases of the AEL schemes, perhaps in order to increase joint stability. Greater activation of the antagonists during the upward phase of the exercise without concomitant alterations in the activation of the agonists would diminish the potentiation of the mechanical variables, potentially explaining the inhibition of the vertical acceleration during the upward phase of the lift during the AEL schemes observed in the present study. Future researchers should therefore include the activation of both agonist and antagonist muscles during different loading schemes to establish potential changes in the coordination of the muscles during the bench press exercise.

We were unable to establish evidence of potentiation of the either the mechanical variables or agonist muscle activation during the upward phase of the AEL caused by the greater loads during the downward phase in the present study. Previous authors have noted that the effectiveness of AEL protocols is likely to be predicated on minimal disruption to the natural mechanics of the movement [10]. Although weight releasers represent a practical and cost-effective means of applying AEL, the potential for these devices to interrupt the movement as they release from the barbell towards the end of the downward phase during the bench press exercise has been highlighted by others [13]. Ojasto and Häkkinen [12] noted alterations in the action of the elbow joint as a result of AEL administered with weight releaser devices. An inspection of the trajectories of the barbell in the sagittal plane during repetitions performed under the different loading schemes used in the present study are revealing. Specifically, comparing the trajectories under the 30AEL and 30T loading schemes (Figure 1A) it can be seen that there are large differences occurring around the transition from the downward to the upward phases, particularly along the anteroposterior axis. These differences occur at a time when the weight releaser devices are detaching from the barbell. Interestingly, there appeared to be little difference in the trajectories under the 80AEL and 80T loading conditions (Figure 1B). Therefore, there was a greater disruption of the lifting technique, as identified by the trajectory of the barbell, during the low AEL scheme used in the present study where the difference between the loads applied during the downward and upward phases was largest. Such disruptions may diminish any enhancement of the musculotendinous units resulting from the accentuated downward load. Indeed, the average RFD recorded during the downward phase of the 30AEL scheme used in the present study was higher than that produced in the other schemes but it also displayed the greatest variation. This greater variation in RFD may have resulted from disruptions in technique experienced by a number of the participants caused by the detachment of the weight releaser devices, thereby negating significantly greater RFD values during the low AEL condition for the overall group. It is noteworthy that Sheppard and Young [19] who demonstrated potentiation of the mechanical variables during the upward phase of the bench throw as a result of AEL schemes administered with weight releasers performed the exercise in a smith machine whereby the potential disruption of the barbell motion as the devices detach was minimized.

The mechanical analysis used in the present study allows for the identification of a number of issues that could confound the effects of AEL protocols. For example, an analysis of the mean vertical force during the downward phases of the AEL schemes demonstrates that neither protocol produced a load that was equivalent to 100% 1RM during the entirety of the phase. This was due to the requirement that the weight releasers detach prior to the completion of the downward phase. Moreover, the mean vertical force during the descent of the barbell was actually significantly different between the two AEL protocols ($\bar{x}$_Diff_: 388 N; ES: 1.40). Specifically, the force during the 80AEL scheme was equivalent to a load of approximately 118 kg (90% 1RM for the present participants), while that during the 30AEL scheme was equivalent to a load of approximately 80 kg (60% 1RM) despite both schemes using the same initial load (100% 1RM). The lower mean load experienced during 30AEL compared to 80AEL is due to the proportion of the downward phase with a lower load (30% 1RM vs. 80% 1RM) after the weight releasers had detached from the barbell. Previous researchers have suggested that stronger participants require greater loads applied during the downward phase in order to potentiate the mechanical aspects of the bench throw exercise [19]. It is possible that the lower load experienced during the downward phase of the 30AEL condition was insufficient to successfully potentiate the active musculature in the group of participants used in the present study.

The mean vertical velocity during the downward phase of 30T was significantly greater than that during 30AEL ($\bar{x}$_Diff_: 0.18 m/s; ES: 0.87). Since the mean vertical force applied to the barbell during the downward phase of the 30T condition was lower than that during 30AEL both produced comparable mean power outputs. However, the differences in the velocity of the barbell’s descent may have obscured any mechanical advantage produced by the AEL loading scheme. Previous researchers have demonstrated that greater downward velocities resulted in enhanced mechanics during the subsequent upward phases of both bench press and back squat exercises [20,21]. It is possible that the greater velocity attained during the downward phase of the 30T condition in the present study resulted in a potentiation of the active musculature or a greater storage and utilization of strain potential energy during the subsequent upward phase that was able to offset any benefit accrued from the greater initial loading experienced during the 30AEL scheme. There were no restrictions placed on the lifting technique in the present study in order to ensure that the participants were not unduly constrained. However, future researchers should consider controlling barbell velocity during the downward phase of the movement when investigating the effects of AEL protocols using the bench press exercise.

## 5. Conclusions

Neither the low (100% 1RM load during the downward phase, 30% 1RM load during the upward phase) or the high (100% 1RM load during the downward phase, 80% 1RM load during the upward phase) AEL protocols used in the present study were able to effectively enhance the mechanics or agonist muscle activation during the upward phase of the bench press exercise. However, the administration of AEL through the use of weight releaser devices may disrupt the lifting technique and also produce loads during the descent of the barbell below those expected, particularly during the low AEL scheme where the contrast in the loads is largest, thereby diminishing any benefits.

## Figures and Tables

**Table 1:** Kinematic data collected during the downward and upward phases of the bench press performed under two accentuated eccentric loading schemes and two traditional loading schemes. Values are means ± standard deviations.

Kinematic Variable	Loading Scheme				Statistical Analyses	
	30AEL	80AEL	30T	80T	Main Effects (*p* < 0.05)	Interactions (*p* < 0.05)
Downward displacement (m)	0.34 ± 0.03	0.34 ± 0.04	0.36 ± 0.04	0.34 ± 0.04	Low loads > High loads Upward > Downward	Scheme × Lifting phase Load × Lifting phase
Upward displacement (m)	0.42 ± 0.04	0.38 ± 0.05	0.40 ± 0.04	0.37 ± 0.05		
Downward time (s)	1.20 ± 0.63	1.18 ± 0.57	0.76 ± 0.25	1.17 ± 0.24	High loads > Low loads Downward > Upward	Scheme × Load interaction Load × Lifting phase Scheme × Load × Lifting phase
Upward time (s)	0.49 ± 0.08	1.02 ± 0.11	0.45 ± 0.08	0.95 ± 0.10		
Downward velocity (m/s)	0.36 ± 0.19	0.34 ± 0.13	0.54 ± 0.22	0.31 ± 0.08	Traditional > AEL Low loads > High loads Upward > Downward	Scheme × Load Load × Lifting phase Scheme × Load × Lifting phase
Upward velocity (m/s)	0.90 ± 0.18	0.38 ± 0.05	0.94 ± 0.18	0.39 ± 0.07		
Downward acceleration (m/s^2^)	7.60 ± 3.85	4.54 ± 1.65	6.02 ± 2.60	3.55 ± 0.52	Low loads > High loads	Scheme × Lifting phase
Upward acceleration (m/s^2^)	7.03 ± 2.16	3.67 ± 1.05	7.55 ± 2.63	3.67 ± 0.80		

Note: 30AEL is the accentuated eccentric loading protocol using a load equivalent to 100% 1-repetition maximum (1RM) during the downward phase and a load equivalent to 30% 1RM during the upward phase; 80AEL is the accentuated eccentric loading protocol using a load equivalent to 100% 1RM during the downward phase and a load equivalent to 80% 1RM during the upward phase; 30T is the traditional loading protocol using a load equivalent to 30% 1RM during the downward and upward phases; 80T is the traditional loading protocol using a load equivalent to 80% 1RM during the downward and upward phases.

**Table 2:** Kinetic data collected during the downward and upward phases of the bench press performed under two accentuated eccentric loading schemes and two traditional loading schemes. Values are means ± standard deviations.

Kinetic Variable	Loading Scheme				Statistical Analyses	
	30AEL	80AEL	30T	80T	Main Effects (*p* < 0.05)	Interactions (*p* < 0.05)
Downward force (N)	773 ± 260	1161 ± 294	413 ± 88	1052 ± 252	AEL > Traditional High loads > Low loads Downward > Upward	Scheme × Load Scheme × Lifting phase Load × Lifting phase Scheme × Load × Lifting phase
Upward force (N)	425 ± 87	1062 ± 253	422 ± 89	1061 ± 253		
Downward RFD (N/s)	7956 ± 4758	7639 ± 2541	7173 ± 3886	7086 ± 2926	None	None
Downward power output (W)	251 ± 79	361 ± 109	224 ± 95	315 ± 60	High loads > Low loads Upward > Downward	Load × Lifting phase
Upward power output (W)	390 ± 90	408 ± 87	398 ± 104	417 ± 105		
Downward work (J)	263 ± 84	401 ± 103	150 ± 29	364 ± 88	AEL > Traditional High loads > Low loads	Scheme × Load Scheme × Lifting phase Load × Lifting phase Scheme × Load × Lifting phase
Upward work (J)	181 ± 34	407 ± 101	174 ± 36	392 ± 102		

Note: 30AEL is the accentuated eccentric loading protocol using a load equivalent to 100% 1-repetition maximum (1RM) during the downward phase and a load equivalent to 30% 1RM during the upward phase; 80AEL is the accentuated eccentric loading protocol using a load equivalent to 100% 1RM during the downward phase and a load equivalent to 80% 1RM during the upward phase; 30T is the traditional loading protocol using a load equivalent to 30% 1RM during the downward and upward phases; 80T is the traditional loading protocol using a load equivalent to 80% 1RM during the downward and upward phases; RFD is the rate of force development.

**Table 3:** Average activation of the pectoralis major and long head triceps brachii muscles during the downward and upward phases of repetitions performed under the four different loading schemes. Values are means ± standard deviations.

Muscle	Lifting Phase	Loading Scheme				Statistical Analyses	
		30AEL	80AEL	30T	80T	Main Effects (*p* < 0.05)	Interactions (*p* < 0.05)
Pectoralis major (µV)	Downward	168 ± 102	241 ± 148	92 ± 66	211 ± 160	AEL > Traditional High loads > Low loads Upward > Downward	Scheme × Load Scheme × Lifting phase
	Upward	250 ± 160	372 ± 258	241 ± 176	376 ± 269		
Triceps brachii (µV)	Downward	209 ± 65	251 ± 85	106 ± 71	200 ± 157	High loads > Low loads Upward > Downward	Scheme × Lifting phase Scheme × Load × Lifting phase
	Upward	283 ± 157	453 ± 216	326 ± 240	470 ± 231		

Note: 30AEL is the accentuated eccentric loading protocol using a load equivalent to 100% 1-repetition maximum (1RM) during the downward phase and a load equivalent to 30% 1RM during the upward phase; 80AEL is the accentuated eccentric loading protocol using a load equivalent to 100% 1RM during the downward phase and a load equivalent to 80% 1RM during the upward phase; 30T is the traditional loading protocol using a load equivalent to 30% 1RM during the downward and upward phases; 80T is the traditional loading protocol using a load equivalent to 80% 1RM during the downward and upward phases.
